# Finished Genome Sequence of the Unicellular Cyanobacterium *Synechocystis* sp. Strain PCC 6714

**DOI:** 10.1128/genomeA.00757-14

**Published:** 2014-07-31

**Authors:** Matthias Kopf, Stephan Klähn, Björn Voss, Kurt Stüber, Bruno Huettel, Richard Reinhardt, Wolfgang R. Hess

**Affiliations:** a Genetics and Experimental Bioinformatics, Faculty of Biology, University of Freiburg, Freiburg, Germany; b Max Planck-Genome-Centre, Cologne, Germany

## Abstract

*Synechocystis* sp. strain PCC 6714 is a unicellular cyanobacterium closely related to the popular model organism *Synechocystis* sp. strain PCC 6803. A combination of PacBio SMRT and Illumina GAIIx data results in a highly accurate finished genome sequence that provides a reliable resource for further comparative analyses.

## GENOME ANNOUNCEMENT

*Synechocystis* sp. strain PCC 6714 (from here on *Synechocystis* 6714) is a unicellular cyanobacterium and closely related to the popular model organism *Synechocystis* sp. strain PCC 6803 (*Synechocystis* 6803). Despite the large number of recently sequenced cyanobacterial genomes (1), there is still no finished genome available for comparative studies with *Synechocystis* 6803. Recent studies based on a draft genome of *Synechocystis* 6714 have already revealed the potential for comparative analysis by explaining its lower salt tolerance (2) as well as differences in two clustered regularly interspaced short palindromic repeats (CRISPR) associated proteins (CRISPR-*cas*) loci (3). *Synechocystis* 6714 has also been investigated for various other unique aspects (4–7).

For SMRT sequencing, high-quality genomic DNA was isolated using the CTAB protocol (8). Libraries were prepared according to the large SMRTbell gDNA protocol (Pacific Biosciences) with 10 kb insert size. Genomic DNA was sequenced with a PacBio RS II platform yielding 134,034 reads with an average length of 3,818 nt. The reads were *de novo* assembled with HS HGAP Assembly version 2 (Pacific Biosciences), resulting in 7 contigs with a 97-fold average coverage. Three of the contigs could be identified as assembly artifacts and were removed. In order to maximize sequence quality, the publicly available Illumina GAIIx-based draft sequences of *Synechocystis* 6714 (2) were fragmented into pieces of 30 nt and mapped against the SMRT-based assembly using the short read mapper segemehl (version 0.1.7-403) (9). All mismatches, insertions, and deletions were corrected in favor of the highly accurate draft assembly sequences. The final contigs were checked for circularization and overlapping ends were trimmed. Gene prediction and annotation were done with RAST (10).

The finished genome of *Synechocystis* 6714 consists of 4 circular contigs. The largest contig represents the chromosome (3.5 Mb) while the other three contigs represent the plasmids pSYLA (109 kb), pSYLB (104 kb), and pSYLC (41 kb). The annotation revealed 3,770 protein-coding sequences, 42 tRNAs, two rRNA clusters, and three loci of CRISPR-*cas* genes located on the plasmids pSYLA and pSYLB. The finding of the putative prophage Psy1, as well as the absence of the *ggtABCD* and *pilA5* genes in the former draft assembly (2), was confirmed in the finished assembly. Additionally, 37 new coding sequences (CDSs), one tRNA, and a second rRNA cluster were detected, compared to the draft genome. The availability of the highly accurate finished genome sequence of *Synechocystis* 6714 provides a reliable resource for future comparative analysis with *Synechocystis* 6803, the most advanced cyanobacterial model.

### Nucleotide sequence accession numbers.

The finished genome sequences have been deposited at DDBJ/ENA/GenBank under the accession no. CP007542 (chromosome), CP007543 (pSYLA), CP007544 (pSYLB), and CP007545 (pSYLC). The versions described in this paper are the first versions.
